# Comparative Effectiveness and Safety of Pertuzumab and Trastuzumab Plus Chemotherapy vs Trastuzumab Plus Chemotherapy for Treatment of Metastatic Breast Cancer

**DOI:** 10.1001/jamanetworkopen.2021.45460

**Published:** 2022-02-28

**Authors:** Wei Fang Dai, Jaclyn M. Beca, Chenthila Nagamuthu, Ning Liu, Claire de Oliveira, Craig C. Earle, Maureen Trudeau, Rebecca E. Mercer, Kelvin K. W. Chan

**Affiliations:** 1 Temerty Faculty of Medicine, University of Toronto, Toronto, Ontario, Canada; 2 Canadian Centre for Applied Research in Cancer Control, Toronto, Ontario, Canada; 3 Ontario Health–Cancer Care Ontario, Ontario, Canada; 4 ICES, Ontario, Canada; 5 Centre for Health Economics and Hull York Medical School, York,; 6 Sunnybrook Health Sciences Centre, Toronto, Ontario, Canada

## Abstract

**Importance:**

To date, limited studies have examined the comparative outcomes of pertuzumab treatment in the real-world setting. End-of-study analyses of the CLEOPATRA trial found median overall survival (OS) of 57.1 months in patients receiving pertuzumab compared with 40.8 months in control patients, a benefit of 16.3 months. However, studies examining the real-world use of pertuzumab have found conflicting results.

**Objective:**

To assess the real-world comparative effectiveness and safety of pertuzumab, trastuzumab, and chemotherapy for patients with metastatic breast cancer in Ontario, Canada.

**Design, Setting, and Participants:**

A population-based retrospective comparative effectiveness research study was conducted. Patients receiving first-line treatments for metastatic breast cancer from January 1, 2008, to March 31, 2018, in Ontario were identified. Data analysis was performed from November 13, 2019, to August 1, 2021. Thirteen patients had received treatment before diagnosis or were not Ontario residents and were excluded from the analysis. Of the remaining 1823 patients identified, 912 received pertuzumab and 911 were control patients. Using propensity-score methods, 579 pairs of patients receiving pertuzumab were matched to those in the control group, resulting in a total of 1158 patients in the final cohort.

**Exposures:**

Patients in the case group received pertuzumab with trastuzumab and chemotherapy and those in the control group received trastuzumab and chemotherapy.

**Main Outcomes and Measures:**

Overall survival (the primary outcome) and hazard ratios (HRs) were calculated using Kaplan-Meier and Cox proportional hazards regression methods. Secondary outcomes included cumulative incidence of safety end points including resource use and adverse events. Follow-up duration was up to 5 years from the start of therapy, with maximum follow-up to March 31, 2019.

**Results:**

Of the 1158 matched patients (579 pairs) receiving pertuzumab and controls, 1151 (99%) were women (mean [SD] age, 58.2 [12.97] years). The median OS was higher in patients receiving pertuzumab (40.2; 95% CI, 35.6-47.8 months) than in the control patients (25.3; 95% CI, 22.8-27.6 months), a median OS improvement of 14.9 months. Pertuzumab was associated with reduced mortality (HR, 0.66; 95% CI, 0.57-0.79). The cumulative incidence of direct hospitalization at 1 year was lower among patients receiving pertuzumab (11.7%) compared with the control patients (19.0%) (*P* < .001).

**Conclusions and Relevance:**

Although the median OS in both the pertuzumab and control groups were shorter in this study than those observed in the CLEOPATRA trial, there appears to be a similar significant OS benefit with pertuzumab in the real-world setting.

## Key Points

**Question:**

What are the real-world comparative outcomes of treatment with pertuzumab, trastuzumab, and chemotherapy for patients with metastatic breast cancer in Ontario, Canada?

**Findings:**

This comparative effectiveness research study including 1158 patients noted that median overall survival was significantly higher in patients receiving pertuzumab than in control patients. At 1 year after starting treatment, the cumulative incidence of direct hospitalization was significantly lower for patients who received pertuzumab than for those in the control group.

**Meaning:**

The findings of this real-world study suggest improved safety and overall survival benefit in patients receiving pertuzumab; these results are similar to those of the pivotal trial.

## Teaser

This cohort study compares outcomes associated with real-world use pertuzumab treatment vs results from a randomized clinical trial in patients with metastatic breast cancer.

## Introduction

Over the past 2 decades, the introduction of anti-*ERBB2* (formerly *HER2* or *HER2/neu*) targeted therapies, such as trastuzumab and, more recently pertuzumab, have greatly improved the outcomes of patients diagnosed with *ERBB2*-positive breast cancer.^1,2,3^ The pivotal CLEOPATRA trial demonstrated that pertuzumab, in addition to trastuzumab and docetaxel chemotherapy, significantly improved progression-free survival and overall survival (OS) compared with trastuzumab and docetaxel alone.^2,3^ The end-of-study results from the CLEOPATRA trial showed that the median OS in the pertuzumab group (57.1 months) was greater than that seen in the control group (40.8 months), with a median OS benefit of 16.3 months (hazard ratio [HR], 0.69; 95% CI, 0.58-0.82), with a landmark 8-year survival of 37% in the pertuzumab group vs 23% in the control group.^4^

Based on the initial CLEOPATRA study, treatment with pertuzumab plus trastuzumab and a taxane was approved by the US Food and Drug Administration on June 8, 2012,^5^ the European Medicines Association on April 3, 2013,^6^ and Health Canada on April 12, 2013.^7^ In Canada, the Canadian Health Technology Assessment Agency reviewed the clinical and economic evidence from the manufacturer and issued a recommendation to conditionally fund pertuzumab publicly based on its clinical benefit, with the condition to improve cost-effectiveness because the initial economic assessment suggested that pertuzumab was not cost-effective at the submitted price.^8^ Following the Canadian Health Technology Assessment Agency recommendation and confidential price negotiations, pertuzumab became universally publicly funded as the standard first-line therapy for *ERBB2*-positive metastatic breast cancer in Ontario, Canada, on November 25, 2013.

Since 2013, studies have shown conflicting results between the real-world survival benefit of pertuzumab and those observed in the CLEOPATRA trial. Although some studies found that real-world OS was comparable to trial findings,^9,10,11^ other studies reported that median OS was lower than that observed in the trial.^12,13,14^ In addition, some studies noted increased cardiac-related adverse events observed in the real-world setting compared with the cardiotoxicity observed in the CLEOPATRA trial.^10,12,15,16^ These studies described mainly the survival of patients who received pertuzumab, without exploring the relative incremental survival benefit when pertuzumab treatment was compared with previous standard treatments used in the real world. The differences between a treatment’s comparative effectiveness in the real world and the comparative efficacy demonstrated in the clinical trial have been reported for other cancer treatments.^17,18,19^ Therefore, we conducted a real-world study to examine the use of pertuzumab plus trastuzumab and chemotherapy compared with trastuzumab and chemotherapy for patients with metastatic breast cancer.

## Methods

### Study Design and Population

We conducted a population-based retrospective comparative effectiveness research study in Ontario, Canada. Adults (age ≥18 years) with an incident diagnosis of breast cancer were identified from the Ontario Cancer Registry (OCR) using *International Classification of Disease for Oncology, Third Edition* codes (C50.0-C50.9). The study cohort was linked to the New Drug Funding Program database held by the Ontario Health-Cancer Care Ontario, to identify patients who received first-line treatments for metastatic breast cancer between January 1, 2008, and March 31, 2018. The New Drug Funding Program provides public reimbursements for patients who are eligible to receive trastuzumab with or without pertuzumab in the metastatic setting. Patients who received pertuzumab, trastuzumab, and chemotherapy in the metastatic setting after the funding date of pertuzumab (November 25, 2013) were considered as pertuzumab cases. Patients who received trastuzumab and chemotherapy in the metastatic setting before the funding date of pertuzumab were considered control patients. Patients were excluded from the cohort if they received pertuzumab before the funding date, received treatment before the OCR diagnosis, or were not an Ontario resident at the time of diagnosis. The index date was the first record of pertuzumab treatment with metastatic intent for pertuzumab cases and the first record of trastuzumab treatment with metastatic intent for control patients. This study was approved by the Sunnybrook Research Ethics Board, with waiver of informed consent because ICES is a prescribed entity and so is able to collect health care data for analysis.

### Data Sources and Reporting

Data were retrieved using multiple linked administrative databases held at ICES, an independent, nonprofit research institute funded by an annual grant from the Ontario Ministry of Health and Ministry of Long-Term Care. The databases used in the study include Registered Persons Database, OCR database, Ontario Health Insurance Plan database, New Drug Funding Program database, Ontario Drug Benefit program, Activity Level Reporting, Canadian Institute of Health Information Discharge Abstract database, Canadian Institute of Health Information Discharge National Ambulatory Care Reporting System, and Same Day Surgery databases. These data sets were linked using unique encoded identifiers and analyzed at ICES. As a prescribed entity under Ontario’s privacy legislation, ICES is authorized to collect and use health care data for the purposes of health system analysis, evaluation, and decision support. Secure access to these data is governed by policies and procedures that are approved by the Information and Privacy Commissioner of Ontario. This study followed the International Society for Pharmacoeconomics and Outcomes Research (ISPOR) reporting guideline and was designed, analyzed, and reported in accordance with the relevant portions of the Strengthening the Reporting of Observational Studies in Epidemiology (STROBE), the RECORD-PE guidelines, as well as the RWE-START structured templates.^20,21^ In accordance with the policies of ICES, small cell counts were suppressed and are reported as less than 6 to limit the risk of patient identification.

### Study Outcomes

The primary outcome was OS, defined as the time from the index date of treatment until death as identified in the Registered Persons Database, a population-based registry. Patient data were censored if the patient remained alive at the end of the follow-up period (March 31, 2019), lost Ontario Health Insurance Plan eligibility, or remained alive at 5 years after the index date. Secondary outcomes of safety end points included resource use and adverse events during treatment. Resource use end points were defined as any hospitalization records or any emergency department visits. Adverse events included those related to cardiotoxicity and febrile neutropenia. A cardiotoxicity-related adverse event was defined using a validated algorithm and included any incident heart failure event resulting in hospital admission (Canadian Institute of Health Information’s Discharge Abstract Database) or 1 ambulatory care diagnosis (Ontario Health Insurance Plan claims database) followed by a second diagnosis (from either source) within 1 year.^22^ Heart failure events were ascertained using *International Classification of Diseases* (*ICD*) diagnostic codes, including *ICD-9* 428 (congestive heart failure) and *ICD-10* 150.0 (congestive heart failure), 150.1 (left ventricular failure), and 150.9 (heart failure, unspecified).^22^ For patients with congestive heart failure at baseline, a cardiotoxicity-related adverse event was defined as an incident hospital admission for heart failure. Febrile neutropenia–related hospital visits were identified using a previously developed algorithm.^23^ The observation window for secondary outcomes was between the index date of treatment and the last dose of trastuzumab plus 21 days.

### Baseline Covariates

The study cohort was linked to administrative data sets to ascertain baseline covariates. Data on age and sex were obtained from the Registered Persons Database. Race and ethnicity data are not routinely collected in the population-based administrative databases in Ontario, Canada. Neighborhood income quintiles, health region of residence (local health integrated network), and rurality status were determined using linkages based on individuals’ postal codes from the Postal Code Conversion File and 2016 Census Statistics Canada data.^24,25^ Baseline comorbidities were characterized by the Charlson comorbidity index score with a 2-year look back from the index year of treatment.^26^ The Charlson comorbidity index score was calculated from hospitalization records from the Canadian Institute of Health Information Discharge Abstract Database and the Canadian Institute of Health Information National Ambulatory Care Reporting System database, excluding the cancer diagnosis. Stage of breast cancer at initial diagnosis as categorized by the Best Stage group, estrogen receptor and progesterone receptor status was identified from the OCR. Prior cancer diagnosis was identified from the OCR and included any cancer diagnosis other than the primary (most recent to index treatment) breast cancer diagnosis occurring within 5 years before the index date. Records of hormonal therapy (letrozole, anastrozole, exemestane, and tamoxifen), lapatinib, bisphosphonate, adjuvant trastuzumab, adjuvant treatment other than trastuzumab, and neoadjuvant chemotherapy treatment were identified from the Ontario Drug Benefit and New Drug Funding Program databases. Radiotherapy records were identified from the Ontario Health Insurance Plan and Activity Level Reporting database obtained from the Ontario Health-Cancer Care Ontario. Prior surgical resections for breast cancer were identified from the Canadian Institute of Health Information’s Discharge Abstract Database.

### Statistical Analysis

Descriptive statistics were used to characterize the study cohort. Continuous variables are reported using mean (SD) or median (IQR). Categorical variables are reported using frequencies and percentages. Baseline differences between the pertuzumab case and control groups in the crude cohort were compared using χ^2^ tests for binary covariates, the Kruskal-Wallis test for categorical variables, *t* tests for normally distributed continuous variables, and Wilcoxon rank-sum tests for nonnormally distributed continuous variables.

Propensity score methods were used to adjust for differences between the pertuzumab case and control groups. Propensity scores were calculated using a logistic regression model including age at index date (continuous), sex (binary), local health integrated network (categorical), neighborhood income quintile (categorical), rurality (binary), Charlson comorbidity index score (categorical), years between cancer diagnosis and index date of treatment (continuous), cancer stage (categorical), prior hormonal therapy (binary), prior bisphosphonate treatment (binary), prior adjuvant trastuzumab (binary), prior adjuvant treatment other than trastuzumab (binary), prior neoadjuvant treatment (binary), prior radiotherapy (binary), prior breast cancer (binary), prior nonbreast cancer (binary), estrogen receptor status (categorical), and progesterone receptor status (categorical). A propensity score–matched cohort between the pertuzumab and control groups was created using 1:1 matching, with a caliper width equal to 0.2.^27^ Standardized differences between the adjusted covariates were calculated, and differences less than or equal to 0.1 are generally considered to represent acceptable balance.^28^

Overall survival was assessed using the Kaplan-Meier method and the difference between the pertuzumab case and control groups was calculated using the log-rank test. Cox proportional hazards regression was used to calculate HRs. Descriptive statistics were used to describe the secondary safety end points. The cumulative incidence function was used to estimate the absolute risk of safety outcomes at 1 year after the index date while accounting for mortality as a competing risk. Fine-Gray competing risk models were calculated to compare the groups.^29^

Three sensitivity analyses were conducted. The first analysis included patients with complete estrogen receptor or progesterone receptor status from the matched cohort. The second analysis excluded patients who did not receive vinorelbine from the matched cohort, which consists of 20% of historical control patients. Vinorelbine with trastuzumab was indicated for metastatic treatment before pertuzumab funding but was not indicated with pertuzumab and trastuzumab treatment. The third analysis included patients younger than 65 years, and a separate propensity score matching was conducted (eTable 1 in the Supplement). Overall survival analysis was conducted for all sensitivity analyses. Findings were considered statistically significant with a 2-sided test at *P* < .05. Statistical analyses were conducted using SAS, version 9.4 (SAS Institute Inc).

## Results

### Study Population

**Figure 1.**
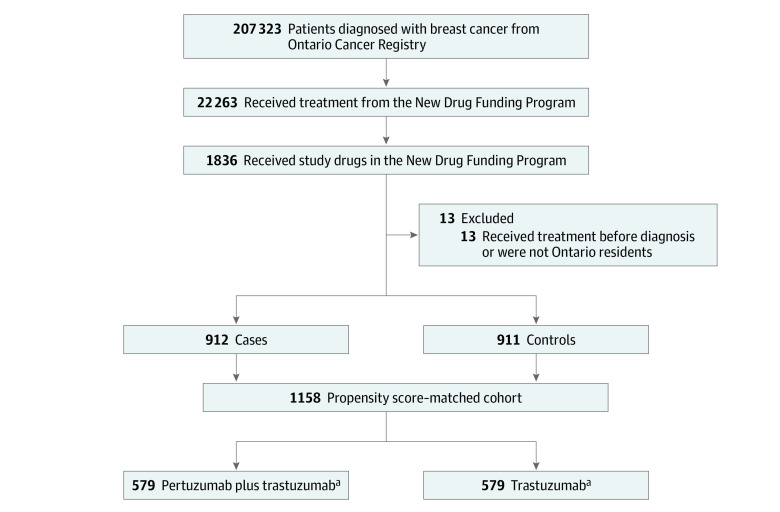
Cohort Creation and Study Design ^a^Both groups received chemotherapy for metastatic intent.

A total of 1836 patients with breast cancer were treated with first-line metastatic treatment between January 1, 2008, and March 31, 2018 (Figure 1). Thirteen patients had received treatment before diagnosis or were not Ontario residents and were excluded from the analysis. Of the remaining 1823 patients identified, 912 were in the pertuzumab group and 911 were in the control group. Using propensity-score methods, 579 pairs of patients receiving pertuzumab were matched to the control patients, resulting in a total of 1158 patients in the final cohort. In the propensity score–matched study cohort, the mean (SD) age was 58.2 (12.97) years, 1151 (99.4%) were women, and 1012 (87.4%) lived in urban regions (Table 1). All baseline variables were balanced in the propensity score–matched cohort.

**Table 1.** Baseline Characteristics of Study Population by Pertuzumab Cases and Controls, Crude and Propensity Score Matched

Covariate^a^	Crude cohort			Propensity score–matched cohort		
	No. (%)		*P* value	No. (%)		Standardized differences
	Pertuzumab (n = 912)	Control (n = 911)		Pertuzumab (n = 579)	Control (n = 579)	
Age at index date, mean (SD), y	57.7 (12.7)	58.1 (12.8)	.50	58.3 (12.5)	58.2 (13.0)	0.01
LHIN, region						
1	38 (4)	52 (6)	.08	30 (5.2)	28 (4.8)	0.02
2	92 (10)	83 (9)		56 (9.7)	54 (9.3)	0.01
3	41 (5)	41 (5)		27 (4.7)	29 (5.0)	0.02
4	81 (9)	73 (8)		52 (9.0)	56 (9.7)	0.02
5	56 (6)	45 (5)		28 (4.8)	27 (4.7)	0.01
6	93 (10)	95 (10)		58 (10.0)	58 (10.0)	0
7	76 (8)	90 (10)		53 (9.2)	48 (8.3)	0.03
8	95 (10)	121 (13)		73 (12.6)	77 (13.3)	0.02
9	88 (10)	92 (10)		56 (9.7)	53 (9.2)	0.02
10	42 (5)	42 (5)		29 (5.0)	29 (5.0)	0
11	136 (15)	103 (11)		70 (12.1)	75 (13.0)	0.03
12	35 (4)	20 (2)		≤20	16 (2.8)	0.01
13	33 (4)	41 (5)		25 (4.3)	22 (3.8)	0.03
14	6 (1)	13 (1)		≤5	7 (1.2)	0.03
Neighborhood income quintile						
1 (lowest)	155 (17.0)	154 (16.9)	.64	99 (17.1)	99 (17.1)	0
2	202 (22.1)	191 (21.0)		135 (23.3)	133 (23.0)	0.01
3	189 (20.7)	195 (21.4)		122 (21.1)	124 (21.4)	0.01
4	174 (19.1)	195 (21.4)		113 (19.5)	114 (19.7)	0
5 (highest)	192 (21.1)	175 (19.2)		110 (19.0)	109 (18.8)	0
Urban residence	801 (87.8)	797 (87.5)	.82	505 (87.2)	507 (87.6)	0.01
Charlson comorbidity index score						
0	505 (55.4)	522 (57.3)	.07	333 (57.5)	327 (56.5)	0.02
1	61 (6.7)	72 (7.9)		41 (7.1)	45 (7.8)	0.03
≥2	16 (1.8)	28 (3.1)		13 (2.2)	17 (2.9)	0.04
No hospitalization	330 (36.2)	289 (31.7)		192 (33.2)	190 (32.8)	0.01
Time between diagnosis to index date, mean (SD), y	2.75 (4.1)	3.10 (3.6)	.06	2.71 (4.14)	2.72 (3.75)	0
Cancer stage at diagnosis						
I	50 (5.5)	36 (4.0)		30 (5.2)	33 (5.7)	0.02
II	177 (19.4)	99 (10.9)	<.001	85 (14.7)	84 (14.5)	0
III	222 (24.3)	156 (17.1)		127 (21.9)	120 (20.7)	0.03
IV	325 (35.6)	284 (31.2)		224 (38.7)	222 (38.3)	0.01
Missing/unknown	138 (15.1)	336 (36.9)		113 (19.5)	120 (20.7)	0.03
Prior status						
Hormonal therapy	136 (14.9)	166 (18.2)	.06	85 (14.7)	93 (16.1)	0.04
Bisphosphonate treatment	88 (9.6)	155 (17.0)	<.001	69 (11.9)	67 (11.6)	0.01
Adjuvant trastuzumab treatment	313 (34.3)	236 (25.9)	<.001	160 (27.6)	160 (27.6)	0
Any adjuvant treatment	209 (22.9)	238 (26.1)	.11	128 (22.1)	118 (20.4)	0.04
Neoadjuvant treatment	122 (13.4)	82 (9.0)	<.01	53 (9.2)	60 (10.4)	0.04
Adjuvant radiotherapy	326 (35.7)	326 (35.8)	.99	187 (32.3)	185 (32.0)	0.01
Breast cancer	66 (7.2)	17 (1.9)	<.001	16 (2.8)	17 (2.9)	0.01
Other cancer	43 (4.7)	31 (3.4)	.16	23 (4.0)	25 (4.3)	0.02
Estrogen receptor^b^						
Negative	189 (39.0)	116 (48.9)	<.001	117 (50.6)	111 (48.3)	0.03
Positive	295 (61.0)	121 (51.1)		114 (49.4)	119 (51.7)	0.02
Progesterone receptor^b^						
Negative	252 (52.3)	152 (64.4)	<.001	149 (64.5)	146 (63.7)	0.01
Positive	230 (47.7)	84 (35.6)		82 (35.5)	83 (36.2)	0

Abbreviation: LHIN, local health integrated network.

^a^ In accordance with the patient privacy policies of ICES, the numbers and percentage values for male and female populations in the data are not reported to avoid the possibility of back calculation of populations less than 5.

^b^ Percentages based on known cases and controls.

### Overall Survival

**Figure 2.**
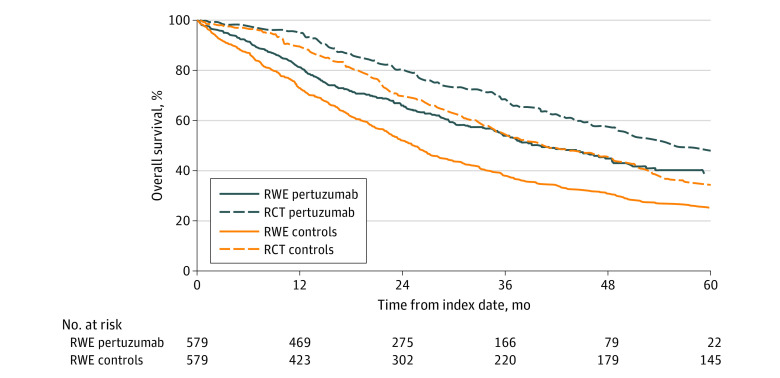
Overall Survival Curves in Propensity Score–Matched Cohort and CLEOPATRA Trial RCT indicates randomized clinical trial; RWE, real-world evidence.

The survival outcomes from the real-world setting compared with the CLEOPATRA trial are presented in Table 2. All patients in the control group had 60.0 months of follow-up; the median follow-up time for the pertuzumab group was 38.5 (95% CI, 35.0-41.4) months. The median OS in the real-world cohort was higher in the pertuzumab group (40.2; 95% CI, 35.6-47.8 months) than in the control group (25.3; 95% CI, 22.8-27.6 months), resulting in a survival benefit of 14.9 months with pertuzumab (Table 2). Figure 2 presents the survival curves from the clinical trial and real-world study. At 1 year, the survival probability was 81% for the pertuzumab group and 73% for the control group. In both the trial and real-world settings, pertuzumab was significantly associated with reduced mortality, with similar HRs (real-world, 0.66; 95% CI, 0.57-0.79; trial, 0.69; 95% CI, 0.58-0.82).

**Table 2.** Comparison of Baseline Covariates and Survival Outcomes Between Trial and Real-World Settings

Variable^a^^,^^b^	No. (%)			
	Real-world		CLEOPATRA Trial^10,12^	
	Pertuzumab (n = 579)	Control (n = 579)	Pertuzumab (n = 402)	Control (n = 406)
Baseline covariates				
Age at index date, median (IQR), y	58 (50-68)	58 (48-67)	54 (22-82)	54 (27-89)
Prior adjuvant or neoadjuvant chemotherapy				
No	341 (59)	317 (55)	218 (54)	214 (53)
Yes	238 (41)	262 (45)	184 (46)	192 (47)
Prior hormonal therapy	85 (15)	93 (16)	106 (26)	97 (24)
Prior adjuvant trastuzumab	160 (28)	160 (28)	47 (12)	41 (10)
Hormone receptor status^c^				
ER-positive, PR-positive, or both	114 (51.1)	119 (53.4)	189 (47.1)	199 (49.1)
ER-negative, PR-negative	109 (48.9)	104 (46.4)	212 (52.9)	196 (50.9)
Survival estimates^d^				
OS, median (95% CI), mo	40.2 (35.6-47.8)	25.3 (22.8-27.6)	57.1 (50-72)	40.8 (36-48)
Follow-up, median (95% CI), mo	38.5 (35.0-41.4)	60.0	99.9 (92.9-106.4)	98.7 (90.9-105.7)
Survival probability, %				
1 y	81	73	94	89
2 y	66	52	81	70
3 y	54	38	68	54
4 y	45	31	58	45
5 y	39	25	49	35

Abbreviations: ER, estrogen receptor; OS, overall survival; PR, progesterone receptor.

^a^ Additional variables reported in the trial: race or ethnic group, region, Eastern Cooperative Oncology Group performance status, disease type at screening, *ERBB2* status, prior anthracycline therapy, and prior taxane therapy. Additional variables in the propensity score model in the real-world study: health region, neighborhood income quintile, rurality, Charlson comorbidity index score, year between diagnosis and treatment date, cancer stage at diagnosis, prior adjuvant radiotherapy, prior breast cancer, and prior other cancer.

^b^ In accordance with the patient privacy policies of ICES, the numbers and percentage values for male and female populations in the data are not reported to avoid the possibility of back calculation of populations less than 5.

^c^ Percentages based on known cases and controls.

^d^ Hazard ratios: real-world setting, 0.66 (95% CI, 0.57-0.79); CLEOPATRA trial, 0.69 (95% CI, 0.58-0.82).

### Sensitivity Analysis

The results of the sensitivity analysis are presented in eTable 2 in the Supplement. In a subcohort of patients with complete estrogen receptor and progesterone receptor records (n = 282), the survival benefit of pertuzumab remained significant (HR, 0.62; 95% CI, 0.45-0.84). In patients who did not receive vinorelbine (n = 924), pertuzumab still showed a significant survival benefit (HR, 0.74; 95% CI, 0.62-0.88). In patients younger than 65 years, pertuzumab also was associated with a significant survival benefit (HR, 0.59; 95% CI, 0.48-0.72), with a median OS of 48.5 months in the pertuzumab group and 26.6 months in the control group. The eFigure in the Supplement presents the survival curves of the younger cohort in the real world overlayed on the survival curves for the overall trial population.

### Safety End Points

Table 3 presents the descriptive summaries of safety end points during treatment. Compared with the control patients, patients in the pertuzumab group were less likely to have an emergency department visit leading to a hospitalization (37.5% vs 32.0%; *P* = .048) or a direct hospitalization (25.6% vs 15.5%; *P* < .001). The 1-year cumulative incidence of a direct hospital visit was lower in the pertuzumab group than the control group (11.7% vs 19.0%; *P* < .001) (Table 3). There were no significant differences between the pertuzumab and control groups for emergency department visits not leading to hospitalization, heart failure–related adverse events, and febrile neutropenia–related adverse events.

**Table 3.** One-Year Cumulative Incidence of Emergency Department Visits, Hospitalizations, and Relevant Adverse Events

Outcome	Events, No. (%)		*P* value	1-y Cumulative incidence, %		*P* value
	Pertuzumab (n = 579)	Control (n = 579)		Pertuzumab (n = 579)	Control (n = 579)	
Any emergency department visit						
Not leading to hospitalization	134 (23.1)	137 (23.7)	.06	21.5	20.5	.75
Leading to hospitalization	185 (32.0)	217 (37.5)	.048	27.8	32.8	.05
Direct hospitalizations	90 (15.5)	148 (25.6)	<.001	11.7	19.0	<.001
Heart failure–related adverse events	29 (5.0)	39 (6.7)	.21	3.9	4.3	.17
Febrile neutropenia–related adverse events	39 (6.7)	48 (8.3)	.32	6.5	8.3	.22

## Discussion

To our knowledge, this is the largest population-based study to date to examine real-world comparative use of pertuzumab vs trastuzumab and chemotherapy compared with trastuzumab and chemotherapy for patients with metastatic breast cancer. In this population-based propensity score–matched study, we found that pertuzumab was associated with improved relative OS in the real world (HR, 0.66), similar to the findings in the CLEOPATRA trial (HR, 0.69). In contrast, the median OS (pertuzumab, 40.2 months; control, 25.3 months) was shorter in the real-world setting than in the trial (pertuzumab, 57.1 months; control, 40.8 months). This resulted in similar survival benefit in the real world (14.9 months) and the trial (16.3 months). Reductions in absolute effectiveness compared with trial findings are arguably an expected finding in real-world studies, where patients may not meet trial eligibility criteria and are typically not free of comorbidities. When compared with the CLEOPATRA trial, patients in this cohort were older (58 vs 54 years) and more likely to have received prior adjuvant trastuzumab treatment. In light of this finding, our study suggests support of the generalizability of the CLEOPATRA trial findings with respect to the incremental benefits of pertuzumab. Moreover, in the real world, patients receiving pertuzumab had fewer emergency department visits requiring hospital admission or direct hospitalizations compared with control patients, suggesting better tumor control with pertuzumab.

Previous studies that have examined real-world outcomes of pertuzumab use found conflicting results. Three multicenter single-arm studies reported better progression-free survival outcomes in the real world (21, 22.8, and 27.8 months)^9,16,30^; however, OS was not examined and the studies may be subject to selection bias based on treatment settings. Three population-based single-arm studies, 2 of which used similar population and data sources as the present study, reported poorer real-world OS results compared with the CLEOPATRA trial (OS, 39.2, 41.8, and 43.0 months).^12,13,14^ In contrast, a recent analysis of US data showed that OS (48.6 months) did not differ significantly in the real-world setting compared with the CLEOPATRA trial, but the authors of this study also discussed the limitations of electronic health records as well as a relatively short median follow-up time.^11^ In addition, in what is, to our knowledge, the only other published comparative real-world analysis, researchers reported a larger survival benefit (real-world HR, 0.47 vs trial HR, 0.69) and similar median OS (51.5 vs 56.5 months from the initial CLEOPATRA trial) for pertuzumab; however, this single-institution study included a small sample of patients who received pertuzumab (50 of 304 patients).^10^

In the real-world setting, we noted a similar 1-year cumulative incidence of cardiac-related adverse events between the pertuzumab and control groups. A previous Ontario study found 1.8 heart failure events that required hospitalization per 100 person-years among patients who received pertuzumab.^12^ The percentage of patients who received pertuzumab and experienced cardiotoxicity-related adverse events in our study was similar to that in other studies,^10,16^ which is also similar to the CLEOPATRA trial result of 3.8%.^15^ The real-world definition of cardiotoxicity-related adverse events we used was different from the definition used by the trial, which defined adverse events as more than a 10% point decline in left-ventricular ejection fraction at baseline, or to less than 50%, or if treatment was required.^15^ In contrast, we defined cardiotoxicity as heart failure events that resulted in either hospitalization or ambulatory care visits. Despite the definition differences, the algorithm for capturing heart failure events has been validated^22^ and can be a useful tool to compare relative adverse events between the pertuzumab and control groups.

### Strengths and Limitations

The strength of this study includes a large sample size owing to the use of population-based administrative health care databases. These databases capture all patients in Ontario, Canada, which comprises a population of approximately 14.5 million people, who would have received the study drug of interest.

The study also has limitations. First, this study is retrospective, which has inherent limitations, such as the lack of randomization. Second, we used a historical comparator from before pertuzumab became available. This approach was used because, after funding became available, there were very few patients who did not receive pertuzumab, and such patients would likely have very different characteristics (confounding by indication), which would preclude useful comparison. The use of a historical baseline and patient comparator may be subject to secular variations, such as changes in treatment practices. In the setting of *ERBB2*-positive metastatic breast cancer, new treatments, such as lapatinib and trastuzumab emtansine, may have also contributed to better survival for the pertuzumab group. Other secular changes, such as improvements in supportive care and management of cardiotoxicity, could also contribute to better outcomes. Although propensity score methods can identify observable confounders, such as baseline demographic and clinical characteristics, propensity scoring has inherent limitations in its ability to account for unobservable confounders.

## Conclusions

The findings of our comparative effectiveness study suggest that the incremental survival benefits seen in the CLEOPATRA trial are being substantially realized in the real-world setting among patients in Ontario, Canada. By comparing patients receiving pertuzumab with historical control patients who received trastuzumab and chemotherapy, we found that the addition of pertuzumab was associated with significant real-world comparative effectiveness in a large population of unselected patients. We also noted no increased cardiotoxicity-related adverse events and fewer direct hospitalizations among patients receiving pertuzumab at 1 year of treatment.

## Notes

Supplement. **eFigure.** Kaplan-Meier Overall Survival Curves in the Propensity Score–Matched Cohort for Patients Who Are Younger Than 65 Years and for CLEOPATRA Trial for Patients of All Ages **eTable 1.** Baseline Characteristics for a Subcohort of Patients Who Are Younger Than 65 Years **eTable 2.** Sensitivity Analysis
