# Supplementary material for: Comparative Effectiveness and Safety of Pertuzumab and Trastuzumab Plus Chemotherapy vs Trastuzumab Plus Chemotherapy for Treatment of Metastatic Breast Cancer
Source: JAMA Netw Open. 2022 Feb 28;5(2):e2145460. doi: 10.1001/jamanetworkopen.2021.45460 (PMC8886524; doi:10.1001/jamanetworkopen.2021.45460)
Supplement: Supplement. — eFigure. Kaplan-Meier Overall Survival Curves in the Propensity Score–Matched Cohort for Patients Who Are Younger Than 65 Years and for CLEOPATRA Trial for Patients of All Ages eTable 1. Baseline Characteristics for a Subcohort of Patients Who Are Younger Than 65 Years eTable 2. Sensitivity Analysis [file jamanetwopen-e2145460-s001.pdf]

Supplementary Online Content

Dai WF, Beca JM, Nagamuthu C, et al. Comparative effectiveness and safety of pertuzumab and trastuzumab plus chemotherapy vs trastuzumab plus chemotherapy for treatment of metastatic breast cancer. *JAMA Netw Open*. 2022;5(2):e2145460. doi:10.1001/jamanetworkopen.2021.45460

eFigure. Kaplan-Meier Overall Survival Curves in the Propensity Score–Matched Cohort for Patients Who Are Younger Than 65 Years and for the CLEOPATRA Trial for Patients of All Ages

eTable 1. Baseline Characteristics for a Subcohort of Patients Who Are Younger Than 65 Years

eTable 2. Sensitivity Analysis

This supplementary material has been provided by the authors to give readers additional information about their work.

© 2022 Dai WF et al. *JAMA Network Open.*

eFigure. Kaplan-Meier Overall Survival curves in the propensity-score matched cohort for patients who are younger than 65 years and for the CLEOPATRA trial for patients of all ages

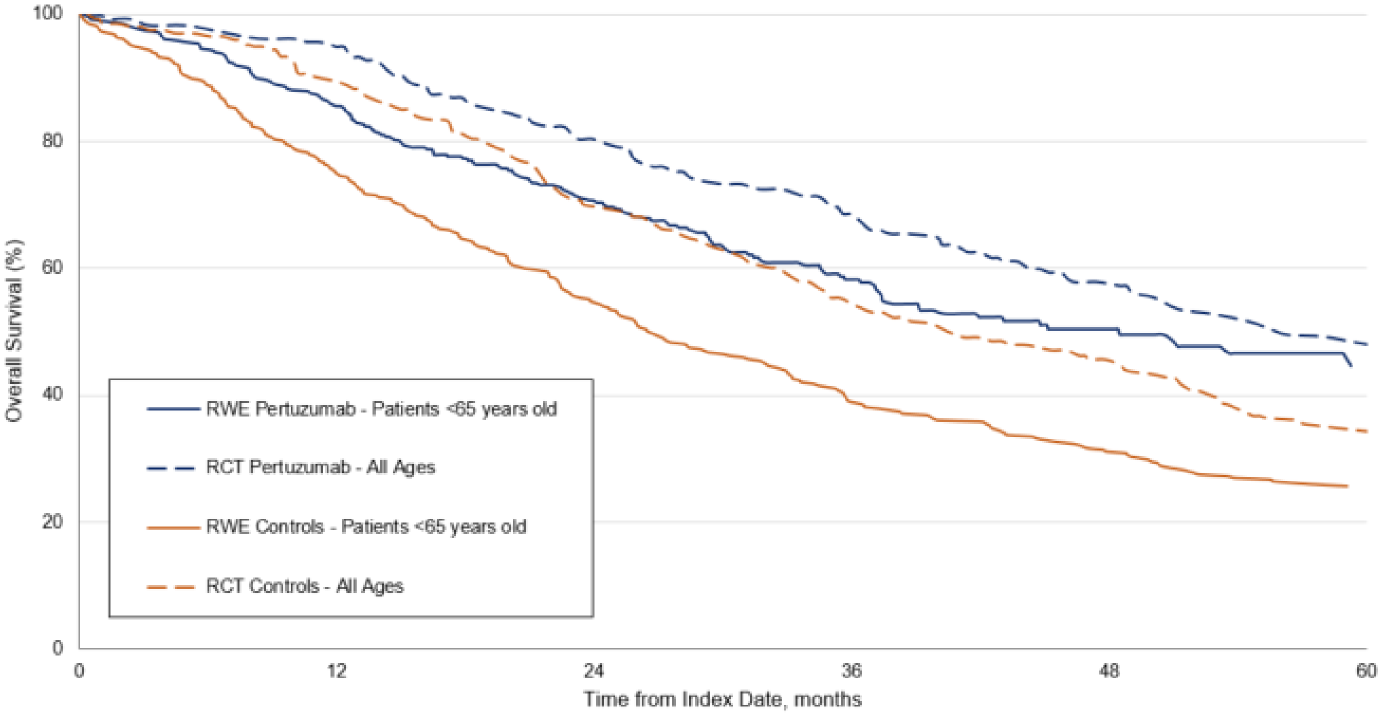

Number at risk:						
RWE Pertuzumab	386	330	202	127	64	17
RWE Controls	386	289	211	150	120	98

© 2022 Dai WF et al. *JAMA Network Open.*

eTable 1: Baseline characteristics for a sub cohort of patients who are younger than 65 years

Covariates	Propensity Score Matched Cohort		
	Pertuzumab (n = 386)	Controls (n = 386)	Std. Diff
Age at index date, Median (range)	52 (47 – 58)	52 (45 – 59)	0.05
LHIN, N (%)			
Region 1	15 (3.9)	13 (3.4)	0.03
Region 2	36 (9.3)	32 (8.3)	0.04
Region 3	21 (5.4)	19 (4.9)	0.02
Region 4	33 (8.5)	31 (8.0)	0.02
Region 5	25 (6.5)	23 (6.0)	0.02
Region 6	43 (11.1)	50 (13.0)	0.06
Region 7	37 (9.6)	36 (9.3)	0.01
Region 8	38 (9.8)	44 (11.4)	0.05
Region 9	44 (11.4)	41 (10.6)	0.02
Region 10	18 (4.7)	19 (4.9)	0.01
Region 11	48 (12.4)	56 (14.5)	0.06
Region 12	<=10	<=10	0.02
Region 13	14 (3.6)	11 (2.8)	0.04
Region 14	<=5	<=5	0.06
Neighbourhood Income Quintile, N (%)			
Quintile 1 (lowest)	67 (17.4)	70 (18.1)	0.02
Quintile 2	77 (19.9)	89 (23.1)	0.08
Quintile 3	81 (21.0)	71 (18.4)	0.07
Quintile 4	81 (21.0)	75 (19.4)	0.04
Quintile 5 (highest)	80 (20.7)	81 (21.0)	0.01
Urban Residence, N (%)	338 (87.6)	337 (87.3)	0.01
Charlson's Score, N (%)			
0	228 (59.1)	224 (58.0)	0.02
1	<=20	<=20	0.02
2+	<=5	<=5	0
No hospitalization	133 (34.5)	139 (36.0)	0.03

© 2022 Dai WF et al. *JAMA Network Open.*

Time between diagnosis to index date, y, Mean ± SD	2.8 ± 4.0	2.8 ± 3.7	0.01
Cancer Stage, N (%)			
I	18 (4.7)	15 (3.9)	0.04
II	44 (11.4)	53 (13.7)	0.07
III	91 (23.6)	82 (21.2)	0.06
IV	151 (39.1)	147 (38.1)	0.02
Missing/Unknown	82 (21.2)	89 (23.1)	0.04
Prior hormonal therapy, N (%)	27 (7.0)	24 (6.2)	0.03
Prior bisphosphonate, N (%)	44 (11.4)	42 (10.9)	0.02
Prior adjuvant trastuzumab, N (%)	128 (33.2)	118 (30.6)	0.06
Prior any adjuvant treatment, N (%)	102 (26.4)	97 (25.1)	0.03
Prior neoadjuvant treatment, N (%)	50 (13.0)	44 (11.4)	0.05
Prior adjuvant radiation, N (%)	144 (37.3)	126 (32.6)	0.1
Prior breast cancer, N (%)	14 (3.6)	13 (3.4)	0.01
Prior other cancer, N (%)	13 (3.4)	13 (3.4)	0
Estrogen Receptor, N (%)[a]			
Negative	76 (49.0)	70 (52.8)	0.04
Positive	68 (51.0)	73 (47.2)	0.03
Progesterone Receptor, N (%)[a]			
Negative	95 (66.4)	92 (64.8)	0.02
Positive	48 (33.6)	50 (35.2)	0.02

[a]Percentages based on known cases and controls

Legend: Std Diff = Standardized Differences

© 2022 Dai WF et al. *JAMA Network Open.*

eTable 2: Sensitivity Analyses

	Pertuzumab Group Median OS, months (95% CI)	Control Groups Median OS, months (95% CI)	Hazard Ratio (95% CI)
**Primary Analysis**			
Propensity-score matched cohort (N = 1,166)	40.2 (35.6 – 47.8)	25.3 (22.8 – 27.6)	0.66 (0.57 – 0.79)
**Scenario A:** Patients with complete ER or PR status (N = 282)	48.7 (35.8 – NR)	27.2 (22.7 – 33.1)	0.62 (0.45 – 0.84)
**Scenario B:** Excluding vinorelbine (N = 924)	40.3 (35.7 – 48.7)	27.3 (25.2 – 32.9)	0.74 (0.62 – 0.88)
**Scenario C:** Patients less than 65 years old (N = 772)	48.5 (37.3 – NR)	26.6 (23.8 - 32)	0.59 (0.48 – 0.72)

Legend: NR = not reached

Note: The threshold of 65 years old was chosen because the median age in the cohort approximates that of the pivotal trial (median age = 54).

© 2022 Dai WF et al. *JAMA Network Open.*
